# D130A variant on Parkinson 22-related CHCHD2 is predicted to have decreased protein movement

**DOI:** 10.17912/micropub.biology.001093

**Published:** 2023-12-22

**Authors:** Hanna J. Jefcoat, Cynthia L. Stenger, Luke Terwilliger, Michele Morris, Olivia Morris

**Affiliations:** 1 Biology, University of North Alabama, Florence, Alabama, United States; 2 Mathematics, University of North Alabama, Florence, Alabama, United States; 3 Computer Science, University of North Alabama, Florence, Alabama, United States; 4 HudsonAlpha Institute for Biotechnology, Huntsville, Alabama, United States

## Abstract

Parkinson’s disease is the second most common neurodegenerative disease which is caused by a lack of dopamine in the brain. Parkinson 22 is a form of Parkinson’s disease caused by variations in the coiled-coil-helix-coiled-coil-helix domain containing 2 (CHCHD2) protein. This study investigates an aspartic acid-to-alanine swap on amino acid position 130 (D130A) of the CHCHD2 protein. We have employed protein modeling, conservation analysis, and molecular dynamics simulations to gain an understanding of the effects of the D130A variant on CHCHD2 protein structure and movement.

**Figure 1. Characterization of D130A:**
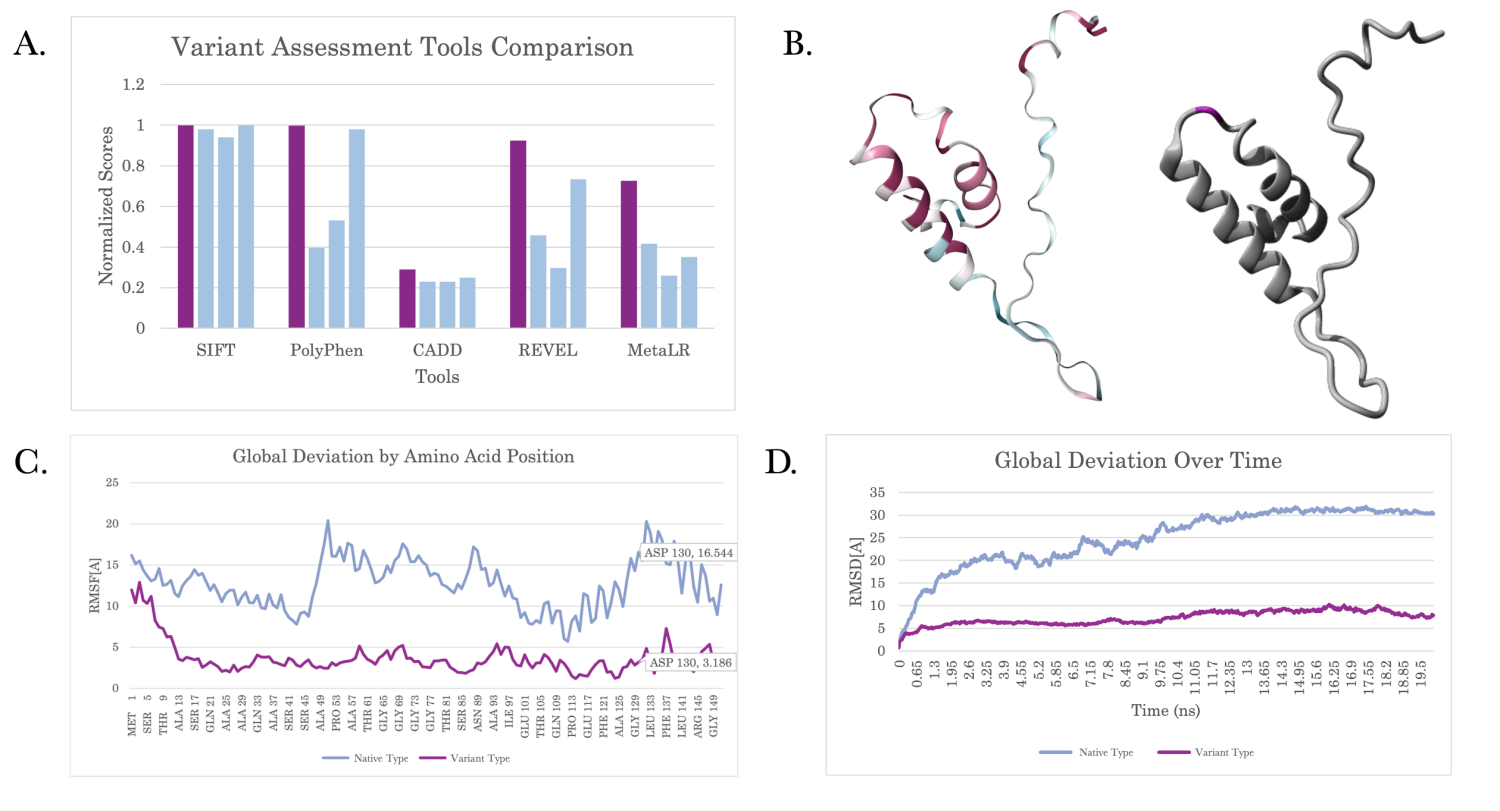
**A)** Analyzed pathogenicity using in silico predictors SIFT, PolyPhen-2, CADD Tools, REVEL, and Meta LR. All scores were normalized for comparison. D130A variant (purple) is ranked as more likely pathogenic than the known pathogenic variants (blue) of CHCHD2 (Ng & Henikoff, 2001; Adzhubei et al., 2010; Rentzsch et al., 2021; Ioannidis et al., 2016; Willer et al., 2010). **B)** Amino acid conservation analysis of the 150 species (left). YASARA homology modeling of CHCHD2 protein (right) (Ashkenazy et al, 2016)(Land et al. 2016). **C)** The carbon alpha root-mean-squared fluctuation (RMSF) of each amino acid throughout the 20 ns simulation. **D)** 20 nanoseconds (ns) of molecular dynamic simulation for the CHCHD2 protein shows the root-mean-squared deviation (RMSD) of the average carbon alpha from the initial structure to each time point of the simulation.

## Description

Parkinson 22 is a form of Parkinson’s Disease caused by mutations on the *CHCHD2* gene; however, few CHCHD2 variants are characterized (Kee et al., 2021) . CHCHD2 is a mitochondrial protein with functions expressed all over the body. This gene is strongly expressed in dopaminergic neurons of the brain, including the substantia nigra. CHCHD2 plays a crucial role in regulating mitochondrial electron flow while also regulating cell migration and differentiation, mitochondrial cristae structure, and apoptosis. Because Parkinson’s disease is caused by a lack of dopamine in the substantia nigra, mutations of the CHCHD2 gene can cause Parkinson 22.

The variant on amino acid position 130 which has a swap from aspartic acid-to-alanine was studied for characterization. All nonpolar amino acid swaps on this gene are classified as pathogenic except for one; D130A is the only polar to nonpolar swap left unclassified (Landrum et al., 2016) . This variant was also found in the CHCH domain, which is a functional region (Apweiler et al., 2004) .

Amino acid position 130 was conserved in 150/150 species, indicating it is likely to serve an important role in the structure and/or function of the CHCHD2 protein (Ashkenazy et al, 2016) . A study on the effect of the D130A swap in a different gene influencing influenza found that this swap had a pathogenic effect (Staller et al., 2021) . The D130A swap on this gene and D130A on CHCHD2 are both located near the end of an alpha helix.

Additionally, the D130A variant has similar pathogenicity scores to the known pathogenic variants. *D130A* was ranked as more likely pathogenic than all other known pathogenic variants. (Ng & Henikoff, 2001; Adzhubei et al., 2010; Rentzsch et al., 2021; Ioannidis et al., 2016; Willer et al., 2010) .

An aspartic acid-to-alanine swap (D212A) in bacteriorhodopsin, a proton-pumping protein used by Archaea, caused the protein to become sensitive to light and stop proton pumping (Mogi et al., 1988) . This finding is consistent with the results of the CHCHD2 D130A molecular dynamics simulations. The molecular dynamics simulations showed that this variant greatly reduced protein movement, indicating the D130A is likely to negatively affect protein function.

According to these findings, the D130A variant of CHCHD2 has a high likelihood of being pathogenic. D130A is a polar to non-polar swap, which is the same as the known pathogenic variants. Many pathogenicity predictors indicate D130A as being pathogenic, and it is ranked as more pathogenic than the known pathogenic variants as shown in Figure 1 . The swap is located in the CHCH functional region and is conserved in 150/150 species. There was a significant decrease in protein movement in the variant type; an RSMD of 13.36Å was measured at D130A with movement across the entire protein. This reveals that D130A potentially has a significant effect on protein function.

## Methods

*CHCHD2* was chosen for study because it is the only gene associated with Parkinson 22 (Landrum et al., 2016) . A variant of unknown significance (VUS) from CHCHD2 was chosen from analyzing different pathogenicity predictors (SIFT, PolyPhen-2, CADD Tools, REVEL, and Meta LR). All scores were normalized for comparison. Slow homology protein models of both the native type and variant type of the protein were constructed from a FASTA file retrieved from Uniprot. These models were used to run molecular dynamics simulation (mds) using YASARA protein modeling to assess how the variant affects protein functionality.
